# Association of Alcohol Consumption with Perception of Attractiveness in a Naturalistic Environment

**DOI:** 10.1093/alcalc/agv096

**Published:** 2015-08-16

**Authors:** Olivia M. Maynard, Andrew L. Skinner, David M. Troy, Angela S. Attwood, Marcus R. Munafò

**Affiliations:** 1 MRC Integrative Epidemiology Unit (IEU), University of Bristol, Bristol, UK; 2 UK Centre for Tobacco and Alcohol Studies, School of Experimental Psychology, University of Bristol, Bristol, UK

## Abstract

**Aims:**

To investigate the relationship between objectively-assessed alcohol consumption and perception of attractiveness in naturalistic drinking environments, and to determine the feasibility and acceptability of conducting a large-scale study in these environments.

**Methods:**

Observational study conducted simultaneously across three public houses in Bristol, UK. Participants were required to rate the attractiveness of male and female face stimuli and landscape stimuli administered via an Android tablet computer application, after which their expired breath alcohol concentration (BrAC) was measured.

**Results:**

Linear regression revealed no clear evidence for relationships between alcohol consumption and either overall perception of attractiveness for stimuli, for faces specifically, or for opposite-sex faces. The naturalistic research methodology was feasible, with high levels of participant engagement and enjoyment.

**Conclusions:**

We found no evidence for a relationship between alcohol consumption and perception of attractiveness in our large-scale naturalistic study. Our study is important given the large sample size, the successful translation of an experimental, laboratory-based paradigm to a naturalistic drinking environment and the high level of public engagement with the study. Future studies should use similarly ecologically-valid methodologies to further explore the conditions under which this effect may be observed and identify the mechanisms underlying any relationships.

## INTRODUCTION

Excessive alcohol consumption is linked to unsafe sexual behaviours (Rehm *et al.*, 2012). This relationship may at least in part, be mediated by increased perceived attractiveness of others after alcohol consumption, a relationship colloquially termed the ‘beer-goggles effect’. Laboratory experiments have found some evidence for this effect: participants in alcohol conditions as compared with placebo conditions rated facial stimuli of opposite-sex individuals more attractive in some (Parker *et al.*, 2008; Attwood *et al.*, 2012) but not all laboratory experiments (Neave *et al.*, 2008). However, these experiments also observed increased attractiveness ratings for same-sex faces among heterosexual participants (Parker *et al.*, 2008) and for landscape stimuli (Attwood *et al.*, 2012). This finding calls into question the mechanism underlying any possible effect, as it suggests that this effect may not be specific to opposite-sex faces and may instead reflect a general positivity bias after consuming alcohol, at least when tested in the laboratory. Indeed, laboratory experiments, conducted under controlled conditions and where a relatively low dose of alcohol is administered, have limited ecological validity and are far removed from settings where unsafe behaviours associated with alcohol consumption typically occur.

Evidence for a relationship between alcohol consumption and perception of attractiveness has been observed in naturalistic drinking environments, with increased alcohol consumption (defined either by self-report or expired breath alcohol levels) related to higher perception of attractiveness for individuals of the opposite sex, in some (Jones *et al.*, 2003; Johnco *et al.*, 2010; Lyvers *et al.*, 2011) but not all (Gladue and Delaney, 1990) studies. A number of naturalistic studies have also observed a ‘closing-time effect’, whereby faces of fellow patrons become more attractive towards closing time, an effect not necessarily related to the consumption of alcohol (Pennebaker *et al.*, 1979; Nida and Koon, 1983; Gladue and Delaney, 1990; Madey *et al.*, 1996). Despite the ecological validity of these studies, each has important methodological limitations, such as a lack of an objective measure of alcohol consumption (Pennebaker *et al.*, 1979; Gladue and Delaney, 1990; Madey *et al.*, 1996; Jones *et al.*, 2003), using different participants across different time points (Madey *et al.*, 1996; Pennebaker *et al.*, 1979), or asking participants to rate the faces of fellow patrons, rather than controlled face stimuli (Pennebaker *et al.*, 1979; Madey *et al.*, 1996; Johnco *et al.*, 2010).

The present study had two aims: (i) to investigate the feasibility of conducting a large-scale study in a naturalistic drinking environment, and (ii) to address the limitations with previous studies and further investigate the relationship between objectively-assessed alcohol consumption and perception of attractiveness in naturalistic drinking environments using an attractiveness rating task similar to that used in previous laboratory experiments (Parker *et al.*, 2008; Attwood *et al.*, 2012).

## METHODS

### Design and overview

This observational study investigated the relationship between self-administered alcohol consumption and perception of attractiveness. This study was conducted simultaneously across three public houses in Bristol, UK. Participants were required to rate the attractiveness of male and female face stimuli and landscape stimuli, after which their expired breath alcohol concentration (BrAC) was measured. Ethics approval was obtained from the University of Bristol Faculty of Science Research Ethics Committee (1103147001).

### Participants

Participants were male and female patrons at the three public houses. Each of these public houses is a member of the same chain (Dawkins Ales) and has similar prices, drinks on sale and patron demographics. Participants were required to be able to give informed consent, as judged by the researcher, and were recruited opportunistically whilst they were at the public house. The study was also promoted through social media.

### Materials

Stimuli for the attractiveness task were those used previously in laboratory experiments (Parker *et al.*, 2008; Attwood *et al.*, 2012) and comprised 40 photographic facial stimuli (20 male and 20 female, mean age 21 years) and 20 landscape stimuli of natural scenes. The male and female face stimuli were photographs of undergraduate students at the University of Bristol. To provide approximate matching for attractiveness between male and female faces, each individual was the current heterosexual partner of one of the other individuals in the opposite-sex face set (Parker *et al.*, 2008). Participants rated each of these 60 images on a 7-point scale, ranging from ‘very unattractive’ to ‘very attractive’. Participants also rated the extent to which they believed they were intoxicated on a 7-point scale, completed the Biphasic Alcohol Effects Scale (BAES) (Martin *et al.*, 1993) and indicated whether they thought alcohol influences sexual arousal (i.e. alcohol expectancies) with a single item Yes/No question. BrAC was measured using an alcohol breathalyser (AlcoDigital 3000, Draeger).

### Procedure

Testing was conducted between 5 pm and 11 pm over two consecutive weekends (Friday and Saturday) during the Bristol Food Festival in May 2014. Patrons were handed information sheets about the study whilst they were at their bar table and, after reading the information sheet, those who wished to participate moved to a separate table to provide consent and participate. To ensure the accuracy of the BrAC reading (which requires a minimum of 20 min to elapse between the last sip of an alcoholic beverage and an accurate reading being taken), participants were asked to abstain from consuming alcohol for 5 min before participating and for the duration of the study (∼15 min). The BrAC reading was then taken at the end of the testing procedure.

The attractiveness rating task was delivered via an Android tablet computer, using a bespoke Android application, and further information about the study was presented on the tablet before participants provided informed consent and recorded their age and sex. Stimuli were presented in a block design, with the order of presentation of the three stimulus blocks (male faces, female faces and landscapes) and the individual stimuli within each block randomized. On completion of this task, perceived intoxication, alcohol expectancies, BrAC, the hour of participation and the name of the public house were recorded. Participants provided their email address and 2 weeks after the end of testing a follow-up email was sent to all participants with debriefing information, the results of a prize draw (all participants had the opportunity to win one of three £50 shopping vouchers) and a short questionnaire regarding participants' experiences of participating in the study.

### Statistical analysis

Associations between alcohol consumption (as measured by BrAC) and attractiveness ratings were assessed using linear regression, treating both BrAC and attractiveness ratings as continuous variables. First, attractiveness ratings for the three image types (male faces, female faces and landscapes) for each participant were averaged to assess the relationship between BrAC and all ratings of attractiveness. Second, associations between BrAC and attractiveness ratings for face stimuli (an average of ratings for male faces and female faces) and landscapes were assessed in separate models. Finally, the relationships between BrAC and attractiveness ratings of opposite- and same-sex faces were analysed in separate models. Therefore, for each analysis, data comprised each participants' BrAC reading and their mean attractiveness rating for each stimulus category. All analyses were conducted both with and without adjustment for age, sex, public house and alcohol expectancies of the effect of alcohol on sexual arousal. Effect sizes are reported as *R*^2^ values, and for adjusted analyses these represent the size of the effect size attributable to BrAC only. In all analyses, unstandardized B coefficients represent the change in attractiveness ratings per one point increase in BrAC (µg/100 ml). We conducted a power calculation which indicated that, in order to detect an effect size of *r =*0.22 for our main outcome of interest, equivalent to that observed in our previous laboratory study (Parker *et al.*, 2008), we would require 163 participants to achieve 80% power at an alpha level of 5%. As we were also interested in exploring the feasibility of our naturalistic design, we planned to recruit as many participants as possible within the designated testing period. We also assessed the ‘closing-time effect’ in exploratory analyses using linear regression to examine the association between attractiveness ratings and the hour at which the participant completed the experiment, treating both as continuous variables. Again, all analyses were conducted both with and without adjustment for age, sex, public house and alcohol expectancies of the effect of alcohol on sexual arousal. Unstandardized B coefficients represent the change in attractiveness ratings per 1 h increase in time of testing. All analyses were conducted in SPSS Statistics (IMB, Version 21).

## RESULTS

### Characteristics of participants

Three hundred and eleven individuals participated. Approximately equal numbers of participants were recruited from the three public houses (*n* = 115, 100 and 96). Participant characteristics, including age, BrAC, perceived intoxication and scores on the BAES subscales are shown in Table 1 and all were normally distributed. Thirty-five participants had not consumed any alcohol at the time of testing as determined by BrACs of 0 µg/100 ml. The average attractiveness of the three stimulus types was as follows: landscapes, M = 4.10, SD = 0.73; female faces, M = 3.14, SD = 0.87; male faces M = 3.84, SD = 0.78. We recruited a larger number of participants than that specified by the sample size calculation, which meant we ultimately had 90% power to detect an effect size of *r* = 0.18, equivalent to our previous study, and 80% power to detect an effect size of *r* = 0.16.

**Table 1.** Characteristics of participants

	Males (*n* = 171)	Females (*n* = 140)	Overall (*n* = 311)
Age (years)	32 (12)	31 (11)	31 (12)
BrAC (µg/100 ml)	22.53 (17.02)	17.90 (14.68)	20.44 (16.15)
Perceived intoxication	3.13 (1.31)	2.94 (1.41)	3.05 (1.36)
BAES stimulated	36.71 (14.27)	36.21 (16.71)	36.49 (15.39)
BAES sedated	23.01 (12.98)	20.74 (12.92)	21.99 (12.98)

Values represent means (standard deviation).

BrAC, Breath Alcohol Concentration; BAES, Biphasic Alcohol Effects Scale.

### Alcohol consumption and perception of attractiveness: ‘beer-goggles effect’

**Fig. 1.**
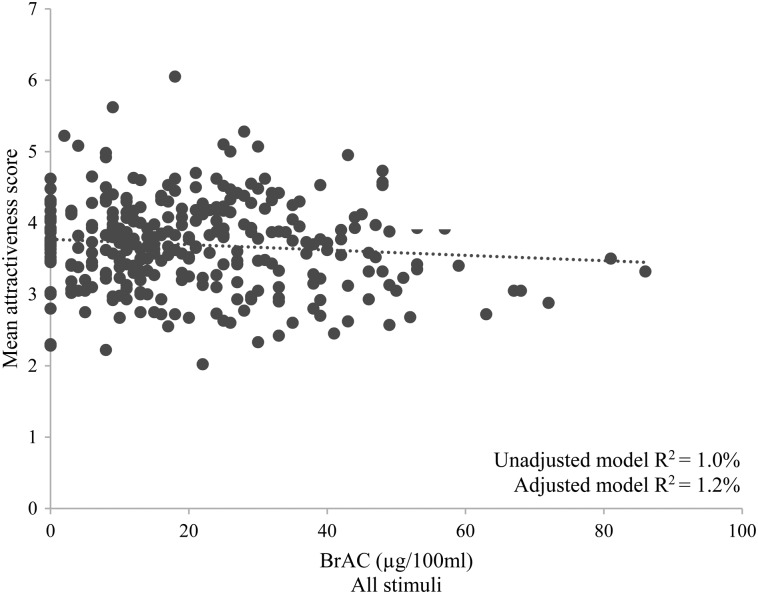
Associations between BrAC and attractiveness ratings for all stimuli.

**Fig. 2.**
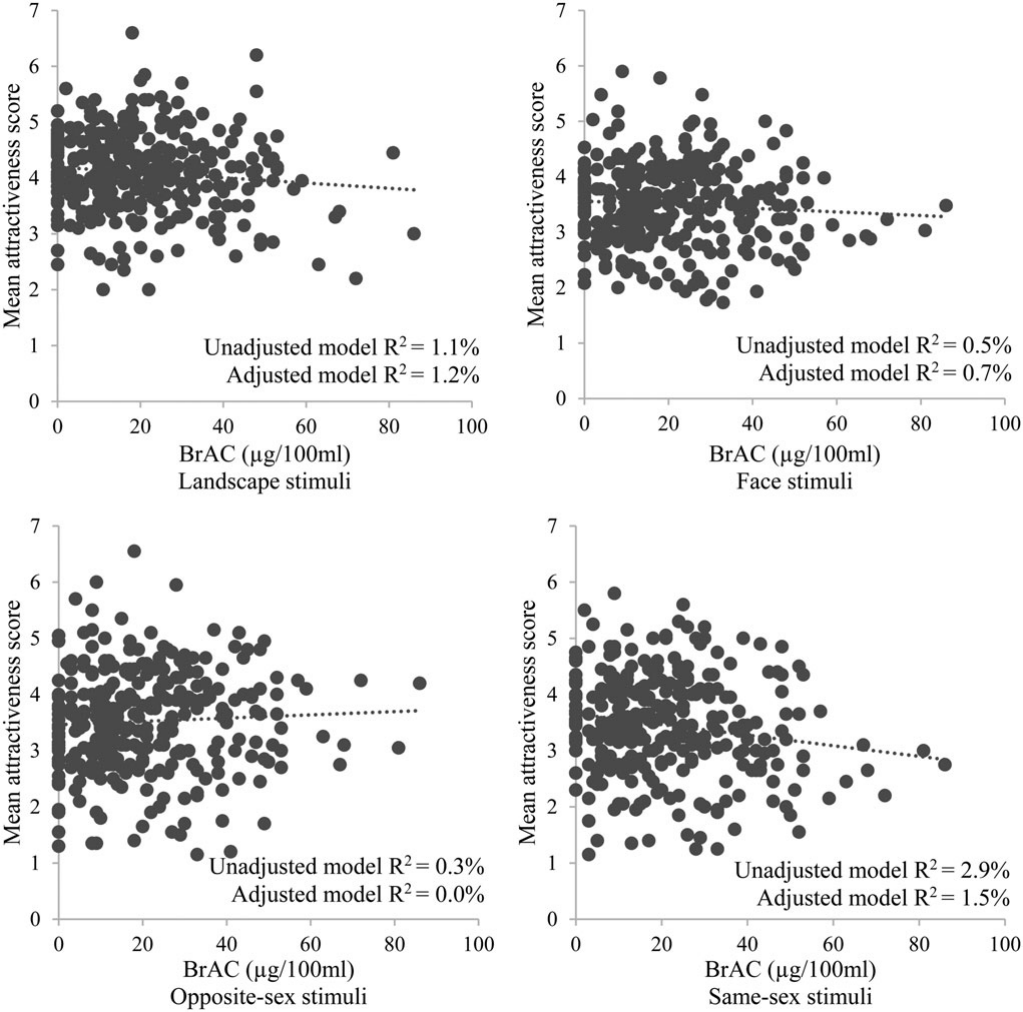
Associations between BrAC and attractiveness ratings for each of the stimuli types.

As shown in Table 2 and Figs. 1 and 2, there was some evidence for a negative relationship between BrAC and attractiveness ratings for all stimuli (adjusted B = −0.004, 95% CI = −0.009 to +0.000, *P* = 0.054, *R*^2^ = 1.2%), landscapes (adjusted B = −0.005, 95% CI = −0.010 to +0.000, *P* = 0.050, *R*^2^ = 1.2%) and all faces (adjusted B = −0.004, 95% CI = −0.009 to +0.001, *P* = 0.140, *R*^2^ = 0.7%). There was evidence for a *negative* relationship between BrAC and attractiveness of same-sex faces in both unadjusted and adjusted analyses (adjusted B = −0.007, 95% CI = −0.013 to −0.001, *P* = 0.022, *R*^2^ = 1.5%). There was an indication that this effect was stronger among male (adjusted B = −0.009, 95% CI = −0.016 to −0.001, *P* = 0.032, *R*^2^ = 2.7%) as compared with female (adjusted B = −0.005, 95% CI = −0.014 to +0.004, *P* = 0.285 *R*^2^ = 0.8%) participants. There was little evidence for a relationship between attractiveness ratings and BrAC for opposite-sex faces in both unadjusted (*R*^2^ = 0.3%) and adjusted models (*R*^2^ = 0.0%), and no clear differences in the relationship between attractiveness ratings and BrAC for male (*R*^2^ = 0.2%) and female (*R*^2^ = 0.0%) participants in adjusted models. All but the unadjusted B coefficient for opposite-sex faces were negative.

**Table 2.** Associations between BrAC and attractiveness ratings

	Attractiveness rating Mean (SD)	Unadjusted				Adjusted			
		B	95% CI		*P*	B	95% CI		*P*
BrAC^a^									
All stimuli	3.70 (0.62)	−0.004	−0.008	+0.001	0.085	−0.004	−0.009	+0.000	0.054
Faces	3.49 (0.73)	−0.003	−0.008	+0.002	0.201	−0.004	−0.009	+0.001	0.140
Landscapes	4.10 (0.73)	−0.005	−0.010	+0.000	0.066	−0.005	−0.010	+0.000	0.050
Opposite-sex faces	3.53 (0.91)	+0.003	−0.003	+0.009	0.372	−0.001	−0.007	+0.005	0.786
Opposite-sex faces—females	3.07 (0.85)	+0.001	−0.009	+0.011	0.844	+0.001	−0.009	+0.011	0.817
Opposite-sex faces—males	3.90 (0.78)	−0.002	−0.009	+0.005	0.599	−0.002	−0.009	+0.005	0.595
Same-sex faces	3.46 (0.89)	−0.009	−0.015	−0.003	0.002	−0.007	−0.013	−0.001	0.022
Same-sex faces—females	3.78 (0.78)	−0.004	−0.013	+0.005	0.381	−0.005	−0.014	+0.004	0.285
Same-sex faces—males	3.21 (0.89)	−0.009	−0.017	−0.001	0.025	−0.009	−0.016	−0.001	0.032
Time of testing^b^									
All stimuli	3.70 (0.62)	−0.021	−0.058	+0.015	0.245	−0.028	−0.065	+0.009	0.135
Faces	3.49 (0.73)	−0.030	−0.072	+0.012	0.164	−0.033	−0.077	+0.010	0.132
Landscapes	4.10 (0.73)	−0.004	−0.047	+0.039	0.848	−0.170	−0.061	+0.026	0.428
Opposite-sex faces	3.53 (0.91)	−0.031	−0.084	+0.023	0.258	−0.038	−0.088	+0.011	0.125
Same-sex faces	3.46 (0.89)	−0.029	−0.081	+0.022	0.263	−0.028	−0.079	+0.022	0.265

^a^Unstandardized B coefficient represents change in attractiveness ratings per one point increase in BrAC (µg/100 ml). Adjusted model includes adjustment for age, sex, public house and alcohol expectancies.

^b^Unstandardized B coefficient represents change in attractiveness ratings per 1 h increase in time of testing. Adjusted model includes adjustment for BrAC, age, sex, public house and alcohol expectancies.

Exploratory analyses examined whether there were differences in the effect of alcohol consumption on ratings of attractiveness for unattractive and attractive stimuli. To examine this, stimuli were split into ‘attractive’ and ‘unattractive’ groups using a median split of attractiveness ratings. Linear regressions found no evidence that alcohol consumption differentially affects attractiveness ratings of unattractive as compared with attractive stimuli.

### Time of measurement and perception of attractiveness: ‘closing-time effect’

Exploratory analyses indicated that there was no clear evidence for an association between ratings of attractiveness for any of the stimuli and the time at which they participated in the experiment (see Table 2).

### Feasibility of naturalistic design

Sixty-four participants (21% of the total) contacted 2 weeks after the study conducted the post-study questionnaire. Thirteen of these participants (4% of the total and 20% of respondents) reported that they came to the public house specifically to participate in the study. The results of this questionnaire are presented in Table 3.

**Table 3.** Results of post-study questionnaire

Question	Rating scale	Responses
To what extent did you mind not drinking for the duration of the experiment?	5-point scale: ‘I minded a lot’ (1)–‘I did not mind at all’ (5)	77% answered 5
To what extent did you find using the tablet computer easy to use?	5-point scale: ‘Very difficult to use’ (1)–‘Not at all difficult to use’ (5)	77% answered 5
How interesting was the study?	4-point scale: ‘Not at all’ (1)–‘Very’ (4)	37% answered 4 97% answered 3 or 4
How enjoyable was the study?	4-point scale: ‘Not at all’ (1)–‘Very’ (4)	38% answered 4 91% answered 3 or 4

In addition, when asked the open question ‘What in particular did you like about this event?’ participants reported that it was ‘interesting’, ‘sociable’ and ‘fun’. Other participants commented that the study was enjoyable, noting that it added an additional social element to the pub visit (e.g. ‘It was social, provided a fun talking point for me and my friends at the pub and was interesting to take part in’; ‘Could be incorporated into a social event, added something extra to a standard pub trip’). Other participants noted that it was interesting to participate in a study in a naturalistic environment (e.g. ‘that research was being conducted “in the field”, making it seem relevant’).

## DISCUSSION

In this large-scale naturalistic study, we find no clear evidence for relationships between alcohol consumption and perception of attractiveness for stimuli overall, for faces specifically or for opposite-sex faces. There was evidence that higher alcohol consumption was associated with finding faces of the same-sex *less* attractive, which has not previously been observed, but until independently replicated this observation should be treated with caution. In exploratory analyses we did not observe a relationship between the time at which participants completed the study and perception of attractiveness, providing no evidence for the ‘closing-time effect’.

The beer-goggles effect has previously been observed in laboratory experiments (Parker *et al.*, 2008; Attwood *et al.*, 2012) but despite using a comparable task in the present study, we did not replicate this effect. An important difference between these laboratory experiments and the present naturalistic study is the self-administration of alcohol here, as compared with random allocation to alcohol or placebo conditions in laboratory experiments. There was a high level of variation in BrAC in the present study, with a mean BrAC of 20 µg/100 ml and 35 participants sober at time of testing. This mean BrAC arguably reflects a lower level of alcohol intoxication than in laboratory experiments where participants are administered 0.4 g/kg of alcohol (Parker *et al.*, 2008; Attwood *et al.*, 2012). It is possible that alcohol may only affect perception of attractiveness at a higher level of alcohol consumption, which was not observable with the naturalistic design used here. Our study was not powered to detect differences between participants with different levels of alcohol intoxication.

We observed no evidence for the closing-time effect in our exploratory analyses. Previous research has suggested a number of different mechanisms for this effect, including: (i) psychological reactance to the concept of leaving a bar alone (Brehm, 1966), (ii) mere exposure to the faces (Johnco *et al.*, 2010), (iii) alcohol expectancy (Fromme *et al.*, 1999), (iv) impaired recognition of facial symmetry (Halsey *et al.*, 2010, 2012) and (v) alcohol myopia (Steele and Josephs, 1990). The first two of these mechanisms assume that fellow patrons are being rated, rather than photographs, and Gladue and Delaney (1990) provide evidence that this effect is only observed when participants are rating the attractiveness of fellow patrons as opposed to photographs of faces. Indeed, the use of standardized photographs in the present study is a key difference from previous naturalistic studies. As suggested by Neave *et al.* (2008), both the closing-time and beer-goggles effects may reflect changes in motivation after consumption of alcohol, rather than a perceptual change, as was examined here. Previous research has shown that expectations of the effect of alcohol on sexual arousal can influence perception of attractiveness after suboptimal alcohol priming (Friedman *et al.*, 2005) and although we controlled for these alcohol expectancies, other motivational changes may also occur during alcohol intoxication. Future research should investigate the conditions under which the beer-goggles and closing-time effects are observed and the mechanisms underlying these, in both naturalistic and laboratory environments.

Our study had a number of important strengths, including the largest sample size of any study in this area, which was sufficient to detect a small effect size (*r* = 0.16) with 80% power. We used the same experimental task as that used in previous laboratory experiments, allowing a relatively direct comparison with these experiments, with the additional strength of conducting the study in a naturalistic environment. Furthermore, our methodology was an improvement on those used previously in naturalistic environments, by including standardized face stimuli and objective measurement of alcohol consumption. In addition, our large sample size, the positive feedback from participants and the general engagement from pub landlords, demonstrates that experimentally rigorous studies can be conducted in naturalistic environments where participants are consuming alcohol, with a high level of participant and landlord engagement.

There were some limitations to our design. First, given the public testing space, participants were not asked to report their sexuality which may have reduced the likelihood of observing any effect of alcohol on attractiveness ratings of opposite-sex faces. Similarly, we did not ask about participants' relationship status or level of sexual arousal, each of which may have impacted their ratings of attractiveness. Second, participants were informed at the start of the study that the aim was to investigate the effect of alcohol on perception of attractiveness. Furthermore, unlike in previous laboratory-based experiments, which used a randomized, placebo controlled design (Parker *et al.*, 2008; Attwood *et al.*, 2012), participants in the present study self-selected their level of alcohol consumption. Both of these factors may have introduced demand characteristics. Third, our naturalistic design also meant that the mean level of alcohol intoxication was relatively low. As discussed above, it is possible that we might have seen more pronounced effects had we observed a larger range of BrACs among participants. Finally, although the large sample size was a key strength of our study, this also increased the likelihood of detecting statistical evidence for small associations that may not be clinically relevant. As a result, the findings reported here should be interpreted in the context of the magnitude of the observed associations, and not simply the statistical evidence against the null hypothesis.

In sum, we find no evidence for a relationship between consumption of alcohol and perception of attractiveness. Our study is important given the large sample size, the successful translation of an experimental, laboratory-based paradigm to a naturalistic drinking environment and the high level of public engagement with the study. Future studies should use similarly ecologically-valid methodologies to further explore the conditions under which this effect may be observed and identify whether it is indeed perceptions of attractiveness which change after consumption of alcohol, or motivations. A meta-analysis of the existing literature would provide further clarification regarding the strength of evidence supporting the relationship between alcohol consumption and perceptions of attractiveness.

## AUTHORS' CONTRIBUTIONS

All authors conceived and designed the study. A.L.S. programmed the task, O.M.M., D.T. and A.S.A. managed the day-to-day running of the trial. M.R.M. and O.M.M. performed the data analysis. All authors contributed to the interpretation of results. The manuscript was drafted by O.M.M. and D.T. with input from all co-authors. All authors read and approved the final version of the manuscript.

## FUNDING

This work was supported by the Medical Research Council and the University of Bristol (MC_UU_12013/6). The funders had no role in any aspect pertinent to the study. We declare that we have not received support from any companies for the submitted work. As the corresponding author, O.M.M. had full access to all of the data in the study and had final responsibility for the decision to submit for publication. Funding to pay the Open Access publication charges for this article was provided by Research Councils UK.

## CONFLICT OF INTEREST STATEMENT

None declared.
